# Insect medicines for colorectal cancer: A review of mechanisms, preclinical evidence, and future prospects

**DOI:** 10.1097/MD.0000000000041873

**Published:** 2025-03-14

**Authors:** Xinyi Ao, Xin Zhou, Jianqin Liu, Qian Wu, Yanlin Yang, Yali Liu, Weian Hao, Li Li, Kaixuan Wang, Zhi Li

**Affiliations:** a Department of Spleen and Stomach Diseases, the Affiliated Traditional Chinese Medicine Hospital, Southwest Medical University, Luzhou, China; b The Key Laboratory of Integrated Traditional Chinese and Western Medicine for Prevention and Treatment of Digestive System Diseases of Luzhou City, the Affiliated Traditional Medicine Hospital, Southwest Medical University, Luzhou, China; c Department of Gastroenterology, Changhai Hospital, Naval Medical University, Shanghai, China.

**Keywords:** colorectal cancer, insect medicine, pharmacological properties, protein peptides, signal transduction

## Abstract

Colorectal cancer is recognized as the third most prevalent malignant tumor globally. The recommended treatment modalities, including surgery, radiotherapy, and chemotherapy, are frequently associated with severe side effects and high recurrence rates. Cancer experts are actively engaged in a global pursuit of safer and more efficacious treatment strategies for colorectal cancer (CRC). Insect medicine, a unique subset of traditional Chinese medicine, is characterized by their broad spectrum of therapeutic effects, which include antibacterial, anticoagulant, antithrombotic, and sedative actions. Insects are enriched with proteins, peptides, and amino acids. These compounds exhibit pharmacological activities, including anti-tumor effects, inhibition of cancer cell proliferation, induction of apoptosis in cancer cells, anti-inflammatory properties, and immunomodulation. Recent studies have revealed that certain traditional Chinese insect medicines, such as Bombyx Batryticatus, Tubiechong, and Aspongopus chinensis Dalls, demonstrate outstanding therapeutic efficacy in the treatment of CRC. The anti-CRC actions of these insect medicines are potentially mediated through mechanisms involving the Hedgehog and Wnt/β-catenin signaling pathways, as well as immunomodulatory effects. Consequently, these insect medicines are proposed as a potential strategy for CRC treatment.

## 1. Introduction

Colorectal cancer (CRC) ranks as the third most prevalent cancer globally and is the second leading cause of cancer-related mortality.^[1]^ Epidemiological data from the American Cancer Society indicate that the global incidence of CRC stands at 10.7%, with a mortality rate of 9.5%. Significantly, the incidence and mortality rates for men exceed those for women. Projections suggest that the global incidence of CRC may rise by approximately 63% by 2040, relative to 2020.^[2]^ In 2020, colorectal cancer (CRC) cases constituted 12.2% of all cancer diagnoses in China and accounted for 28.8% of the global incidence of this disease.^[3]^ Given these statistics, CRC has emerged as one of the significant challenges in public health. Typical clinical manifestations of CRC include dysfunction of the digestive system, characterized by symptoms such as bloating, abdominal pain, and abnormal stools.^[4]^ As CRC progresses, patients may develop systemic symptoms, including anemia, malnutrition, and fever. The absence of pronounced symptoms in the early stages often results in many individuals being diagnosed at a more advanced stage of the disease. Consequently, early screening is paramount. The American Cancer Society advises that individuals at average risk begin regular screening at the age of 45. Should fecal tests, flexible sigmoidoscopy, or CT colonography yield abnormal results, a colonoscopy is recommended to complete the screening process.^[5]^

The development of CRC is influenced by a combination of genetic, epigenetic, and environmental factors.^[6]^ Throughout the progression of CRC, the intestinal mucosa experiences a progressive transformation from normal tissue to adenoma and subsequently to cancer. Research highlights that crucial oncogenic signaling pathways serve as “initiators” in the process of carcinogenesis. Accumulated genetic mutations within the Wnt/β-catenin, p53, and TGF-β/SMAD pathways disrupt the normal mechanisms of cellular growth, apoptosis, and DNA repair, resulting in the uncontrolled proliferation of atypical and heterogeneous cancer cells.^[7]^ Epigenetics plays a crucial role in regulating gene expression, and research has highlighted gaps between specific gene expression patterns associated with CRC and the lack of corresponding genetic variations. Notably, microsatellite instability, a hallmark of certain CRC molecular subtypes, may arise from epigenetic silencing driven by hypermethylation of the MLH1 gene promoter. Global hypomethylation, which contributes to chromosomal instability, is frequently observed in CRC. Furthermore, miRNAs play a significant role in inhibiting protein expression at the post-transcriptional level, influencing numerous pathways associated with cancer. This modulation impacts virtually every stage of CRC, from its initiation through progression to metastasis.^[8]^ Environmental factors including overweight, obesity, physical activity, and dietary patterns have been demonstrated to be associated with the gut microbiome.^[9,10]^ The gut microbiome and its metabolic byproducts play a role in the pathogenesis of CRC by influencing the host’s immune response and regulating intestinal inflammation. Alterations in the composition of the gut microbiome correlate with CRC risk. The detection of specific microbial markers across diverse populations indicates a potential connection between the composition of the gut microbiome and the risk of CRC.^[11,12]^ The pathogenesis of CRC is complex, influenced by the interplay of genetic, epigenetic, and environmental risk factors.

Current treatment approaches for CRC) encompass both surgical and pharmacological interventions. The choice of surgical intervention depends on the stage and location of the cancer, as well as the patient’s overall health. Early-stage cancers (stage 0 and stage 1 tumors) are typically managed through polypectomy or local excision during colonoscopy. In cases of advanced cancer, partial or total colectomy may be indicated, often including lymph node dissection.^[13]^ However, these procedures carry risks, including the potential for residual cancer and perforation. Pharmacological treatments for CRC encompass radiotherapy, chemotherapy, and targeted therapies.^[14]^ However, these interventions are often costly and associated with substantial side effects. Additionally, recent advances in immunotherapy, particularly the approval of 2 antibodies targeting programmed cell death protein 1, have provided new treatment options for patients with metastatic colorectal cancer (mCRC) exhibiting high microsatellite instability and mismatch repair deficiencies (dMMR)^[15]^ However, these therapies are associated with severe side effects and potential issues related to immune tolerance, underscoring the critical need for the development of safer and more effective treatment modalities.^[16]^

Traditional Chinese medicine, leveraging its natural medicinal properties, exhibits unique benefits in the realm of cancer therapy.^[17]^ Insect medicine, a hallmark of traditional Chinese medical practice, has been employed for millennia to address complex health conditions. Insect medicine is considered to be flesh and blood in Chinese (bodily healing, physical coherence, and equilibrium), Which has distinctive efficacies such as activating circulation to remove blood stasis, removing swelling, fostering tissue regeneration, harmonizing qi and blood, as well as fortifying the body’s fundamental health. Advancements in modern pharmacological research methodologies have facilitated the identification of the active constituents in insect medicines. Studies have shown that small molecule peptides present in centipedes may suppress the proliferation of liver cancer cells by regulating the tyrosine phosphorylation pathway. Extracts derived from leeches are shown to inhibit retinoblastoma by impeding the proliferation of WERI-RB-1 cells.^[18]^ A novel anticancer protein EPS72, purified from tubiechong, has been shown to suppress human lung cancer A-549 cells by inducing apoptotic death characterized by cell detachment.^[19]^ This review consolidates recent findings on the active constituents and potential mechanisms of insect medicines in the fight against CRC. It aims to offer fresh perspectives for devising novel, integrated therapeutic approaches for this malignancy. Insect medicines, known for their multi-target effects, low toxicity, and minimal side effects in oncological treatments, have emerged as a significant resource for developing novel anti-CRC drugs.^[20]^ Owing to their biological similarity to humans, insect medicines exhibit potential advantages in treating CRC, characterized by high absorption and utilization rates in the human body. This may contribute significantly to their efficacy in CRC therapy.^[21]^

## 2. Materials and methods

### 2.1. Sources of literature

This review was conducted through a systematic search of databases such as China National Knowledge Infrastructure (CNKI), WANFANG DATA, PubMed, Google Scholar, and Web of Science. The search spanned literature published from January 1, 2000, to March 31, 2024, aiming to evaluate advancements in the research on the use of insect medicine in treating CRC.

### 2.2. Retrieval methods

The search strategy employed both Chinese and English keywords and their combinations: (“Earthworm” OR “Scorpion” OR “Bombyx Batryticatus” OR “Centipede” OR “Leech” OR “Dilong” OR “Quanxie” OR “Jiuxiangchong” OR “Tubiechong” OR “Jiangcan” OR “Wugong” OR “Shuizhi”) AND (“Tumor” OR “Cancer” OR “Colorectal Cancer” OR “Zhongliu” OR “Aizheng” OR “Jiezhichangai”). A “fuzzy” matching option was selected to enhance the search effectiveness, complemented by manual retrieval to ensure no relevant studies were overlooked. Data analysis was conducted using Excel 2021.

### 2.3. Criteria for literature inclusion and exclusion

#### 2.3.1. Inclusion criteria

2.3.1.1. Required literature includes experimental research on applying single-herb insect medicine in vitro cell lines or animal models for CRC treatment.
2.3.1.2. Research must provide detailed data on the efficacy, pharmacological effects, toxicity, and safety of the insect medicine.
2.3.1.3. Research must be published in peer-reviewed scientific journals.
2.3.1.4. Publications must be authored in either English or Chinese.

#### 2.3.2. Exclusion criteria

2.3.2.1. Opinion pieces, editorials, conference abstracts, or case reports.
2.3.2.2. Studies with insufficient methodological rigor or incomplete result reporting.
2.3.2.3. Literature directly on clinical trials or case studies.
2.3.2.4. Duplicate publications or secondary literature analyses.
2.3.2.5. Research not specifying the insect medicine component or not tested within CRC models.

**Figure 1.**
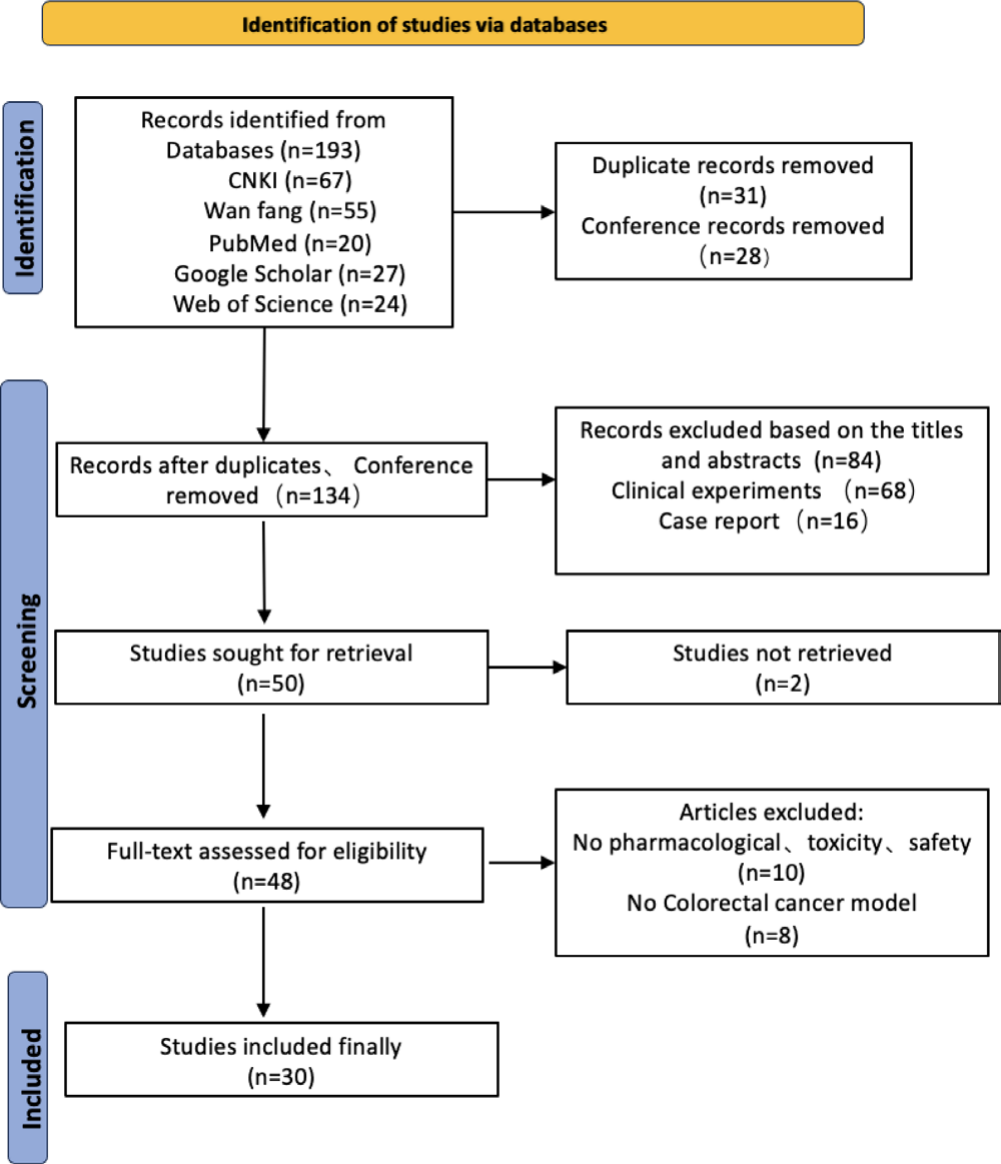
Flow chart of study selection.

Initial screening will involve assessing titles and abstracts to exclude studies that do not meet the inclusion criteria, followed by full-text reviews of potentially relevant studies for further assessment. (Fig. 1 illustrates the flow chart of study selection, providing a visual summary of the steps involved in screening and including studies in this analysis)

## 3. Overview of insect medicine

### 3.1. Historical origins of insect medicine

The history of insect medicine dates back to oracle bone inscriptions from four thousand years ago. These inscriptions include references to medicinal animals such as snakes, musk deer, and rhinoceroses, indicating the early origins of the use of insects, in medicinal practices. In the Spring and Autumn and Warring States periods, ancient literary works, such as the “Classic of Mountains and Seas” and the “Classic of Poetry,” offered detailed accounts of animal-based medicines.^[22]^ The “Shennong Bencao Jing,” regarded as the earliest existing pharmacological treatise, extensively documents various insect medicines. It represents the initial systematization of entomotherapy, providing a crucial foundation for the development of theories and practices in traditional Chinese medicine. During the Eastern Han Dynasty, Zhang Zhong jing used insect medicine to treat conditions such as mental disturbances associated with blood stasis in the lower energizer, acute abdominal pain, and various gynecological disorders. This practice further validated the effectiveness and significance of insect medicine in clinical settings. In the Ming Dynasty, Li Shizhen’s “Compendium of Materia Medica” comprehensively and systematically encapsulated the application experiences of insect medicine, elevating its study to unprecedented levels. This work solidified the role of insect medicine as an indispensable component within the repository of Traditional Chinese Medicine. To this day, insect medicines retain their unique value and demonstrate substantial potential in contemporary medical research and clinical applications.

### 3.2. The characteristics of insect medicine in traditional Chinese medicine

Insect medicines, also called zoological drugs, primarily include dried medicinal animals (excluding internal organs), specific body parts, secretions, excretions, and substances produced in both normal and pathological states. These substances undergo specific processing techniques to form a vital component of TCM therapeutic practice.^[22]^ In Traditional Chinese Medicine theory, the etiology of CRC is attributed to the invasion of toxins, obstruction of qi and blood, phlegm and blood stasis, and the gathering of cancer toxins over time. Cancer toxins are identified as the primary pathological mechanism driving the onset and progression of CRC. Consequently, the core of the treatment strategy focuses on detoxification and anti-cancer therapies.^[23]^ Owing to their potent medicinal properties, insect medicines are considered capable of penetrating deep into the body, directly targeting lesions, and exhibiting the ability to detoxify and remove stasis.^[21]^ Consequently, insect medicines are increasingly being utilized in the treatment of CRC, aiming to disperse the stubborn malignancies associated with tumors.

## 4. Component analysis of insect medicines

Insect medicines encompass a diverse array of types with intricate compositions. The scientific foundations underlying their efficacy and toxicity are not yet fully clarified. Consequently, it is crucial to explore and develop advanced techniques for the effective analysis of active components in these medicines. At present, high-performance liquid chromatography (HPLC), thin-layer chromatography, mass spectrometry, and their associated hybrid techniques are extensively utilized in the analysis of insect medicines.^[24]^ These techniques are favored due to their high sensitivity, specificity, and capability to analyze complex mixtures of compounds. Cai et al^[25]^ successfully developed an HPLC fingerprint for free amino acids in the ultrafine powder of Tubiechong using HPLC, marking 24 common peaks. According to the 2020 edition of the “Chinese Pharmacopoeia,” more than one hundred types of medicinal materials and their formulations, which utilize active components from animal medicines, are qualitatively identified using thin-layer chromatography. Yang et al^[26]^ utilized GC-MS technology to identify 13 free protein amino acids and 3 free non-protein amino acids from scorpion extracts. Chen et al^[27]^ identified 92 proteins in centipede extracts using nanoRPLC-MS/MS. Huang et al^[28]^ identified a novel anticoagulant peptide with a molecular weight of 1790 Da from leech extracts, utilizing affinity chromatography coupled with ultra-high performance liquid chromatography-high resolution mass spectrometry. The peptide sequence is SYELPDGQVITIGNER. The integration of these advanced analytical techniques holds significant scientific importance for the in-depth investigation of the active components in insect medicines. (Please refer to Table 1 for details, Table 1 presents the application of various modern analytical techniques in different insect medicines. Techniques such as GC-MS, LC-MS, and HPLC have been employed to identify a range of compounds, including free proteinogenic amino acids and novel anticancer proteins found in scorpions.^[25,27–37]^)

**Table 1:** The application of modern analytical techniques in insect medicine

No	Insect medicine	Sample type	Analytical techniques	Compounds	reference
1	Scorpion	Lyophilized substances	GC-MS	Thirteen free proteinogenic amino acids (Ile, Phe, Met, Asp, Thr, Tyr, Leu, Val, Ser, Glu, Gly, Pro, Ala) and 3 non-proteinogenic amino acids (Tau, β-Ala, GABA)	^[25]^
2		live specimens	LC-MS	Scorpion toxin family – omegascorpins	^[29]^
3		live specimens	MS	A novel anticancer protein – leptulipin	^[30]^
4	Centipede	live specimens	LC-MS-MS	A novel allergen – Scom5(comprising 210 amino acids)	^[31]^
5		live specimens	HPLC-MS	molecular weight 1018.997 Da peptide sequence RAQNHYCK	^[32]^
6		dried forms	nanoRPLC-MS/MS	Ninety-two proteins	^[27]^
7	Leech	medicinal slices	UPLC- HR- MS	A novel thrombin inhibitor peptide with the sequence SYELPDGQVIGENER	^[28]^
8		live specimens	LC- MS	A novel antiplatelet protein – pigrin	^[33]^
9	Tubiechong	ultrafine powders	HPLC	Six free proteinogenic amino acids(Gly, Arg, Ala, Pro, His, Lys)	^[25]^
10		live specimens	LC- MS	The active peptide – DP-17 with the sequence DAVPGAGPAGCHPGAGP	^[34]^
11	Aspongopus chinensi	live specimens	HPLC	molecular weight 2853.3Da inhibits the proliferation of gastric cancer cells SGC-7901	^[35]^
12		methanol extracts	GC- MS	oleic acid, palmitic acid, z-11-hexadecenoic acid, Threitol, stearic acid, 2-hexenoic acid, 1-(14-methylhexadecanoyl) pyrrolidine	^[36]^
13	Bombyx Batryticatus	extraction solutions	UHPLC-Q-TOF MS	Lumichrome, Demethylated Chlorophyll A p-Coumaroyl Esters 4’-O-β-D-Pyranoglucoside	^[37]^

## 5. Mechanisms of anti-CRC activity in insect medicine and its active components

### 5.1. Types of proteins and peptides

Proteins and peptides, serving as crucial active components in insect medicine.^[38,39]^ (Please refer to Table 2 for details, Table 2 outlines the proteins and peptides identified in insect medicine, listing their relative molecular mass and amino acid sequences. These proteins and peptides have been shown to play critical roles in the therapeutic effects of insect-derived treatments.^[40–60]^), play key biological roles in organisms. Proteins, complex biomolecules found ubiquitously across all organisms, are composed of amino acid residues connected by peptide bonds. They are essential for executing primary cellular functions and maintaining the structural and functional integrity of an organism’s tissues and organs.^[61]^ Peptides, which are shorter amino acid chains linked by peptide bonds, perform diverse functions within organisms. These functions include acting as carriers for drug delivery,^[62,63]^ regulating immune responses,^[64]^ and contributing to disease treatment. Proteins and peptides exhibit anti-cancer potential through various mechanisms. They directly interact with cancer cell membranes, causing membrane disruption and cell death.^[65]^ Additionally, they inhibit tumor growth, angiogenesis, and metastasis, thereby hindering the development and spread of tumors. These substances activate the immune system to combat tumors, thereby enhancing the body’s natural defenses. They also suppress cancer cells by inducing oxidative stress and various forms of cell death.^[66]^

**Table 2:** Protein/peptide in insect medicine

No	Insect medicine	Protein/peptide name	Relative molecular mass/da	Amino acid/peptide sequence	Reference
1	Earthworm	F1	24.6	VVGGSDTTIGQYPHQLSLRVTG	^[40]^
2		F2	26.8	IIGGSNASPGEFPWQLSQTRG	^[40]^
3		F3	28.2	VIGGTNASPGEFPWQLSQQRQ	^[40]^
4		F4	25.4	VIGGTDAAPGEFPWQLSQTR	^[40]^
5		F5	33.1	IVGGIEARPYEFPWQVSVRRKS	^[40]^
6		F6	33	IVGGIEARPYEFPWQVSVRRKS	^[40]^
7		EFE	29.5	RKKGASWFWPWSVKKR	^[41]^
8		D2(8)	23335.14	IVGGYTCGA	^[42]^
9		Band13	15983.48	GVHLTDAEKA	^[42]^
10		Band9	23334.69	GVHLTDAEKA	^[42]^
11		Band7	33317.19	GVVISIANQKGGVG	^[42]^
12		ARSP1	28000	N-terminal 25 Amino Acid Sequence – IIGGTNASPGEFPWQLSQTRGGSHS	^[43]^
13		F-1	535.27	Ac-AMVSS	^[44]^
14		F-2	519.27	Ac-AMVGT	^[44]^
15		VQ-5	519.277	VSSVQ	^[45]^
16		AQ-5	535.218	AMAQQ	^[45]^
17		GGNG2	1598.6	GKCAGQWAlHAAGGNGOH	^[46]^
18		GGNG3	1824.8	PKCSRWAHSCGGGNGOH	^[46]^
19		GGNG1	1756	ARPKCAGRWAIHSCGGGNGOH	^[46]^
20	Scorpion	ANTP	6280	N-terminal 25 Amino Acid Sequence – VRDGYIADDKNCAYFCGRNAYCDDE	^[47]^
21		BMK 9(3)-1	7020	GRDAYIADSENCPYFCGANPN	^[48]^
22		BMK 9(3)2	7037	GRDAYIADSENCPYTCALNP	^[48]^
23		Pi5	3334	VAKCSTSECGHACQQAGCRNSGCRYGSCICV	^[49]^
				GC	
24		Pi6	3126.5	VDACYEACMHHHMNSDDCIEACKNPVPP	^[49]^
25		AEP	8290	N-terminal 25 Amino Acid Sequence – DGYIRGSDNCKVSCLLGNEGCNKECRAYGASYGYCWTVKLAQDCEGLPDT	^[50]^
26	Centipede	scolopin1	2593.9	FLPKMSTKLRVPYRRGTKDYH	^[51]^
27		scolopin2	3017.6	GILKKFMLHRGTKVYKMRTLSKRSH	^[51]^
28		scolopendrin I	4498	/	^[52]^
29		scolopendrasin V	3723.9	YYGGGYKYKHWGCR-NH2	^[53]^
30		scolopendrasin VII	3414.1	FCTCNVKGFNAKNKRGYP	^[53]^
31		LBLP	2757.6	RMKKLGNHK VSCERNTKRCRKAI	^[54]^
32	Tubiechong	ES-termicin	4055.58	ACDFQQCWVTCQRQYSINFISARCNGDSCVCTFRT	^[55]^
33		DP17	1430	DAVPGAGPAGCHPGAGP	^[34]^
34		LL8	NA	LAPAPGTL	^[56]^
35		AR-9	816.3	AVFPSIVGR	^[57]^
36	Bombyx Batryticatus	BB octapeptide	885	AspProAspAlaAspIIeLeuGln	^[58]^
37		BCCI	13,973.63	N-terminal Amino Acid Sequence – VRNKRQSNDD	^[59]^
38	Aspongopus Chinensis Dalls	Cc AMP1	1 997.37	SKITDILAKLGKVLAHV	^[60]^

Since 1983, researchers have identified 48 types of earthworm protein peptides, including their names, relative molecular weights, amino acid sequences, isoelectric points, and biological activities. Notable among these are lumbrokinases, which are fibrinolytic enzymes, as well as antimicrobial peptides and nuclease-like proteins.^[67]^ Arkadiusz Czerwonka et al^[68]^ isolated protein-carbohydrate active molecules (AF) from earthworm coelomic fluid. These molecules exhibited inhibitory effects on CRC cells in vitro while having no impact on the survival and morphology of normal human colon epithelial cells (CCD 841 CoTr). Specifically, AF significantly reduces mitochondrial metabolism in HT-29 cancer cells, disrupts the cell cycle, and induces apoptosis by activating caspase-3 precursors. Additionally, AF inhibits the activity of the human 20S proteasome. Xie et al^[43]^ extracted and identified protein components with anti-tumor activity from the earthworm Eisenia fetida. These protein components demonstrate significant selective inhibitory effects on specific human cancer cell lines, including SY5Y, K562, MGc803, and HeLa. Notably, from these extracts, Apoptosis-Related Serine Protease 1 (ARSP1) was isolated and identified. ARSP1 induces apoptosis in HCT-116 human CRC cells, effectively killing them. Furthermore, in animal models, ARSP1 has been shown to significantly prolong the survival of tumor-bearing mice.

Centipede venom, primarily found in the venomous secretion of centipedes, consists mainly of enzymes and peptides.^[69]^ Antimicrobial peptides in centipede venom not only exhibit lethal activity against bacteria and fungi but also demonstrate inhibitory effects on certain cancer cells.^[70]^ Lee et al^[71]^ discovered that Scolopendrasin VII interacts with phosphatidylserine, a prevalent component on cancer cell surfaces, inducing necrotic cell death. This interaction demonstrates selectivity towards cancer cells, with minimal effects on the viability of non-cancerous cells. Zhao et al^[72]^ discovered that a centipede polysaccharide-protein complex enhances tumor immune responses by downregulating the arachidonic acid (AA) metabolic pathway in tumor-associated macrophages, consequently inhibiting tumor growth in vivo.

Scorpion venom is a complex secretion emitted by entire scorpions, comprised primarily of protein and non-protein components. It possesses the potential to induce paralysis or even death in humans. Recent research has further elucidated that scorpion venom contains a variety of scorpion venom peptides. These peptides are the crucial active components that mediate the venom’s pharmacological actions.^[73]^ He et al^[74]^ discovered that the East Asian scorpion venom toxin (BMK) exhibits an inhibitory effect on human CRC cells Caco-2 within a concentration range of 10 to 40 μg/mL. This inhibitory effect strengthens with increased concentration and extended exposure duration. Furthermore, the mechanism by which BMK inhibits the growth of Caco-2 cells may be associated with its promotion of lymph transformation and its cytotoxic effects.

**Figure 2.**
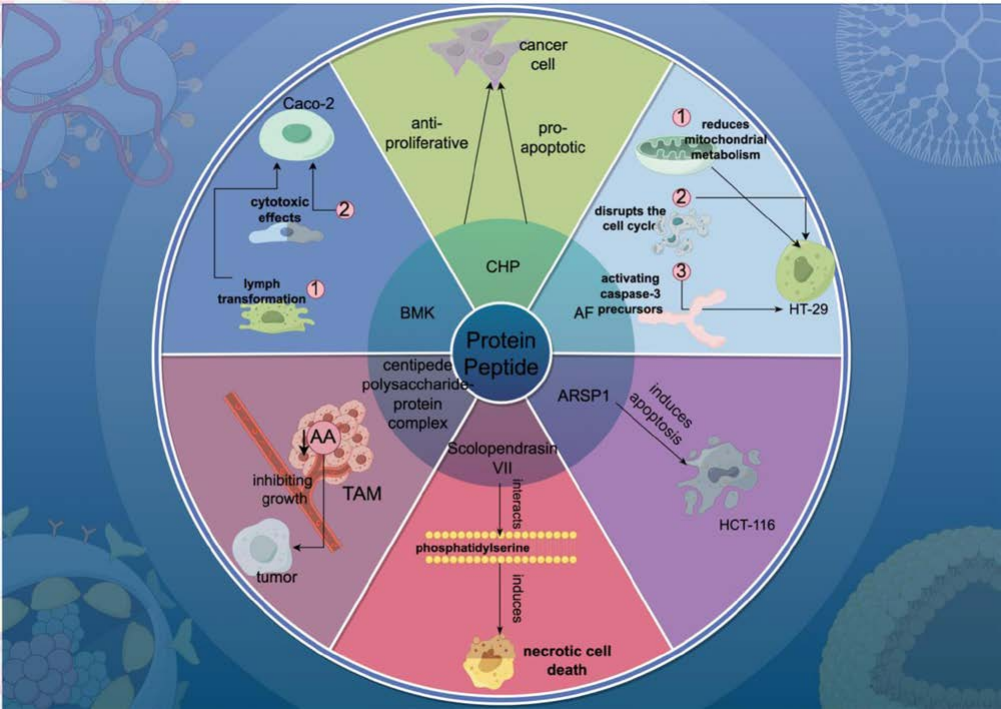
The mechanism of action of protein/peptide in insect medicines.

Tan et al^[75]^ isolated a protein component (CHP) from Aspongopus chinensis Dallas, which exhibits significant anti-proliferative and pro-apoptotic effects on cancer cells in a concentration-dependent manner. Additionally, it demonstrates anti-tumor activity in vivo. (Fig. 2 demonstrates the mechanism of action of various proteins and peptides found in insect medicines, such as earthworm coelomic fluid (AF and ARSP1), centipede venom (Scolopendrasin VII), and scorpion venom (BMK))

### 5.2. Trace elements

Trace elements, such as iron, selenium, magnesium, copper, and molybdenum, though present in minor quantities in insect medicine, play a significant role in its anti-tumor effects.^[76,77]^ They participate in tumor therapy through multiple mechanisms, such as regulating oxidation-reduction reactions, influencing the cell cycle, and activating immune responses.^[77]^ The trace elements iron and copper have been detected in insect medicines, including Tubiechong, Bombyx Batryticatus, scorpions, centipedes, leeches, and Aspongopus chinensis. Iron is a critical driver of ferroptosis, an iron-dependent cell death mechanism. It induces cell death through the promotion of polyunsaturated fatty acids lipid peroxidation, accumulation of iron ions, and formation of lipid peroxides. This mechanism holds potential application value in the treatment of CRC.^[78]^ The regulation of ferroptosis involves various molecular pathways, such as lipoxygenases and long-chain fatty acid CoA synthetase (ACSL4). These pathways increase cellular sensitivity to ferroptosis by integrating polyunsaturated fatty acids into the cell membrane.^[79]^ Additionally, antioxidant defense mechanisms, notably the activity of glutathione peroxidase (GPX4), play a critical role in ferroptosis. Inhibition of GPX4 can be facilitated by specific drugs, such as Erastin and RSL3, which demonstrate potential in curbing the growth and survival of CRC cells.^[80]^

**Figure 3.**
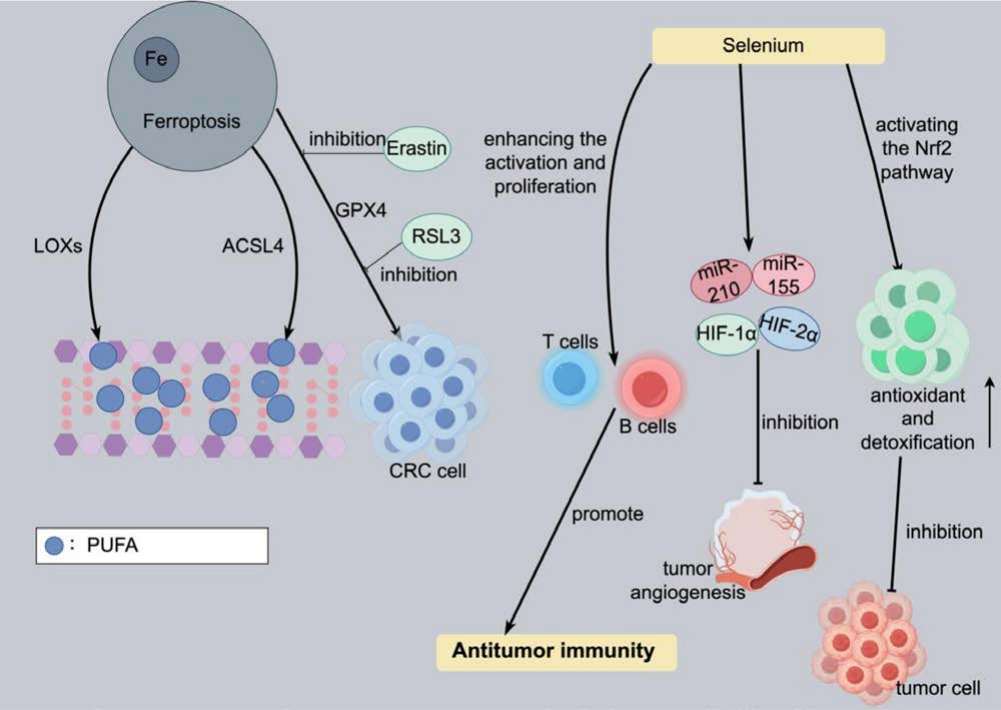
The mechanism of action of trace elements in insect medicines.

Selenium, present in insect medicines like tabanus and earthworms, plays a multifaceted role in tumor therapy. The mechanisms of action of selenium include regulating the immune system, providing antioxidant and cell-protective functions, influencing microRNAs and hypoxia-inducible factors, participating in DNA repair, activating the Nrf2 pathway, and exerting anti-inflammatory effects. By enhancing the activation and proliferation of B cells and T cells, selenium promotes the body’s anti-tumor immune response.^[81]^ As a component of selenoproteins, selenium’s antioxidant properties help mitigate cellular damage induced by free radicals and inhibit tumor growth by shielding cells from oxidative stress.^[82]^ Furthermore, selenium modulates tumor angiogenesis, growth, and drug resistance by regulating microRNAs associated with tumor growth, such as miR-210 and miR-155, and hypoxia-inducible factors, including HIF-1α and HIF-2α.^[83]^ Selenium also contributes to DNA repair mechanisms, enhancing cellular repair capabilities against chemotherapy-induced DNA damage,^[84]^ by activating the Nrf2 pathway, selenium boosts cellular antioxidant and detoxification abilities, thus inhibiting tumor cell growth and survival, and enhancing the efficacy of chemotherapy drugs.^[85]^ Furthermore, selenium’s anti-inflammatory properties aid in reducing inflammatory responses within the tumor microenvironment, further impeding tumor progression.^[86]^ Studies have shown that serum selenium levels below 80 μg/L may elevate the risk of CRC. Conversely, higher concentrations of selenoprotein P (PSELENOP) correlate with a reduced risk of CRC, with particularly significant effects observed in women.^[87]^ Therefore, regulating selenium intake and optimizing selenium status could potentially exert a positive impact on the prevention and treatment of CRC. (Fig. 3 shows the mechanism of action of trace elements present in insect medicines, detailing how they contribute to the therapeutic effects.)

### 5.3. Alkaloids

Alkaloids, a class of nitrogen-containing organic compounds, have been extensively researched for their applications in tumor therapy. These compounds are known to inhibit tumor cell proliferation, induce apoptosis, suppress angiogenesis, block signal transduction, alter the tumor microenvironment, and promote cell differentiation.^[88]^ Four alkaloids have been identified in whole scorpion extracts: 1-stearoyl-glycerol-3-phosphocholine, trigonelline, scorpionine A, and scorpionine B. (Please refer to Table 3 for details, Table 3 summarizes the alkaloids found in insect medicine, including their chemical structures, molecular formulas, and molecular weights. These alkaloids are important for their pharmacological activities.^[89–91]^). Notably, the trigonelline, also present in fenugreek seeds and coffee beans, exhibits significant anti-tumor activity.^[92,93]^ Sun et al^[94]^ demonstrated that trigonelline effectively inhibits the proliferation, migration, invasion, and colony formation of HCT116 and SW480 cells by suppressing the Hedgehog signaling pathway. Furthermore, Li et al^[95]^ discovered that trigonelline significantly inhibits the growth of colorectal tumors both in vivo and in vitro by targeting and suppressing the linc ROR-Wnt/β-catenin signaling axis in CRC.

**Table 3:**
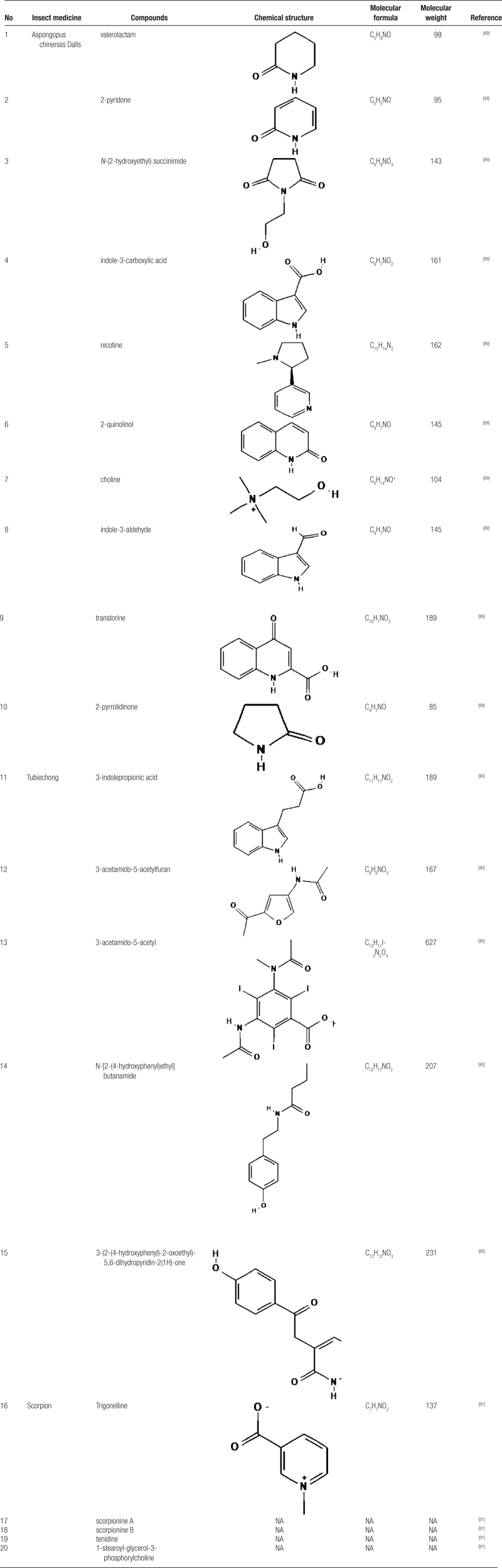
Alkaloids in insect medicine

**Figure 4.**
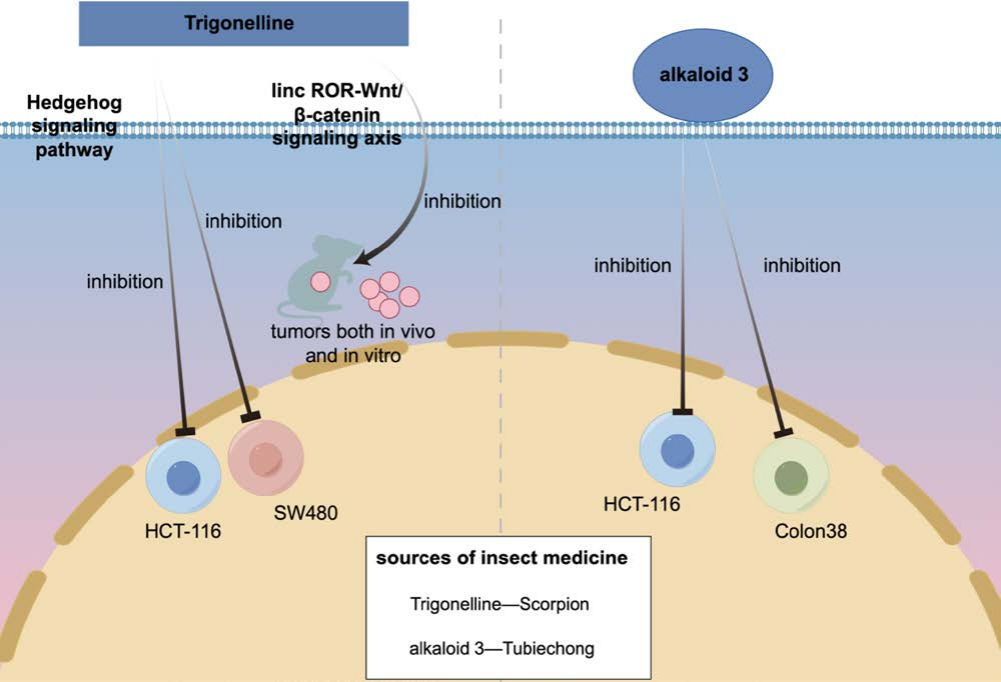
The mechanism of action of alkaloids in insect medicines.

Further studies have identified 21 alkaloids in both Tubiechong and its whole-body ethanol extract. Notably, alkaloid 3, isolated from the ethyl acetate extract of Tubiechong, demonstrated varying degrees of inhibition on Colon38 and HCT-116 cells.^[96,97]^ (Fig. 4 illustrates the mechanism of action of alkaloids in insect medicines, highlighting their roles in the pharmacological activities of these treatments.)

## 6. In vitro, preclinical, and clinical evidence

### 6.1. In vitro studies

Most evidence for insect medicines against CRC arises from in vitro experiments. Earthworm extracts demonstrate selective inhibitory activity against CRC cell lines (HCT-116, HT-29), potentially through apoptosis induction.^[43,68]^

### 6.2. Preclinical studies

Several in vivo studies have further elucidated the anti-CRC potential of insect-based Traditional Chinese Medicines. For instance, extracts from *Eisenia fetida* significantly inhibited tumor growth in xenograft mice, with one isolated protein (ARSP1) inducing apoptosis in CRC cells and prolonging animal survival.^[43]^ Similarly, scorpion venom and centipede-derived peptides have demonstrated notable tumor suppression effects in murine models by modulating immune responses and inducing necrosis.^[72,74]^ (Please refer to Table 4 for details, Table 4 summarizes representative preclinical studies on insect medicines for CRC, detailing the insect source, active components, experimental models, and key anticancer findings.^[43,71,74,75]^)

**Table 4:** Selected insect medicines with anti-CRC activity in preclinical studies

No	Insect medicine	Active component	Model/cell line	Key findings	Reference
1	Earthworm (E. fetida)	ARSP1 protein	HCT-116 xenograft	Induces apoptosis, prolongs survival	^[43]^
2	Scorpion (B. martensii)	BMK toxin	Caco-2 (in vitro)	Inhibits proliferation, promotes lymph transformation	^[74]^
3	Centipede (S. subspinipes)	Scolopendrasin VII	Various tumor lines	Membrane disruption, Selective necrotic cell death	^[71]^
4	Aspongopus chinensis	CHP fraction	H22-bearing mice	Anti-proliferative, proapoptotic in vivo	^[75]^

### 6.3. Clinical studies

Due to our exclusion criteria, direct clinical reports were not included. Limited pilot clinical or case-based studies have suggested potential benefits of insect medicines for CRC,^[21]^ but large-scale randomized controlled trials remain lacking. Further investigation is warranted to confirm efficacy and safety in clinical settings.

## 7. Discussion

In CRC research, insect medicine is gaining attention due to its rich array of bioactive components, including protein peptides, trace elements, and alkaloids. These components have been shown to directly interact with cancer cell membranes, induce cell death, and inhibit tumor growth, angiogenesis, and metastasis through various mechanisms, thus impeding further tumor progression. Research on iron has highlighted its critical role in promoting cancer cell death through iron-dependent mechanisms, known as ferroptosis, providing new perspectives for CRC treatment. Furthermore, these elements also activate the immune system, enhance natural defenses, and combat tumor cells through the induction of oxidative stress and various cell death mechanisms. Although initial research suggests potential anti-tumor effects of these active components, their safety, efficacy, and specific applications in cancer therapy need to be validated through additional clinical trials and mechanistic studies. Such research lays the groundwork for developing novel anti-cancer strategies and guides the direction of future cancer treatment studies.

In Traditional Chinese Medicine, the use of insect medicine in the treatment of CRC has a long history. However, due to concerns over the unknown toxicity profile of insect medicines, it becomes increasingly important to rigorously assess their safety and potential toxicity as modern toxicology research advances. Research indicates that the hepatorenal toxicity of centipedes is significant, and the dosage range linked to liver damage may be between 2 to 7 centipedes per day.^[98–100]^ The primary harmful substances found in Tubiechong are heavy metals and aflatoxins, which can lead to damage to the skin and its appendages, as well as the nervous and circulatory systems. Active components found in Bombyx Batryticatus, including ammonium oxalate and Beauveria bassiana toxin, have been confirmed to be toxic. These substances can induce symptoms such as limb tremors, abdominal pain, and diarrhea, impacting both the nervous and digestive systems. Toxicological research on scorpion venom has demonstrated that it can induce liver and kidney damage, neurotoxic reactions, and respiratory suppression. Consequently, toxicological research is essential in determining the safety of insect medicine for clinical use. This includes identifying potential harmful components, assessing toxicity at various dosages, and elucidating toxicological mechanisms. Considering the multiple active components in insect medicine, interactions among them could result in unforeseen toxic effects. Interactions among components can either enhance or reduce the toxicity of individual elements, making the study of these compound effects vital for evaluating the overall safety of a medication. Furthermore, the potential long-term toxicity is a critical consideration in the safe utilization of insect medicine. While some components might show minimal toxicity in the short term, prolonged use could result in chronic health issues, such as liver or kidney damage.

Literature reviews indicate the clinical dosages for centipedes range from 3 to 5 grams when decocted and 0.6 to 1 gram when administered as a powder; For scorpions, modern clinical usage typically ranges from 3 to 6 grams, with common dosages up to 10 grams; Earthworms and Tubiechong commonly use doses of 3 to 60 grams and 6 to 12 grams, respectively. These figures offer guidelines for dosing insect medicine in the treatment of CRC, with an emphasis on ensuring safety.

## 8. Conclusion and future prospects

This review delves into the advancements in using insect medicine for CRC treatment, with a focus on the anti-tumor mechanisms of its active components. These include inducing apoptosis, inhibiting cell proliferation, blocking angiogenesis, and activating immune responses. The mechanisms span various signaling pathways, including Hedgehog, Wnt/β-catenin, and immune regulatory pathways, offering a molecular foundation for the anti-tumor efficacy of insect medicine.

While existing laboratory studies and preliminary clinical trials provide support for the use of insect medicine, significant challenges remain. The safety, toxicity, and effective dosages require further validation through extensive clinical research. Moreover, the development of efficient extraction and purification techniques for active components, and a clear elucidation of their molecular mechanisms, are imperative. Future research should focus on exploring the interactions and synergistic effects between insect-based Chinese medicine and traditional anti-cancer drugs, and its potential role in comprehensive anti-cancer therapy.

## Author contributions

**Investigation:** Qian Wu, Yanlin Yang, Yali Liu, Weian Hao.

**Supervision:** Zhi Li, Li Li, Kaixuan Wang.

**Writing – original draft:** Xinyi Ao, Xin Zhou.

**Writing – review & editing:** Jianqin Liu.
